# Human Papillomavirus Is Associated With Adenocarcinoma of Lung: A Population-Based Cohort Study

**DOI:** 10.3389/fmed.2022.932196

**Published:** 2022-06-30

**Authors:** Jing-Yang Huang, Chuck Lin, Stella Chin-Shaw Tsai, Frank Cheau-Feng Lin

**Affiliations:** ^1^Department of Medical Research, Chung Shan Medical University Hospital, Taichung, Taiwan; ^2^Institute of Medicine, Chung Shan Medical University, Taichung, Taiwan; ^3^College of William and Mary, Williamsburg, VA, United States; ^4^Superintendents' Office, Tungs' Taichung Metro Harbor Hospital, Taichung, Taiwan; ^5^National Chung Hsing University, Taichung, Taiwan; ^6^Department of Thoracic Surgery, Chung Shan Medical University Hospital, Taichung, Taiwan; ^7^School of Medicine, Chung Shan Medical University, Taichung, Taiwan

**Keywords:** human papillomavirus, lung cancer, adenocarcinoma, cohort study, big data

## Abstract

**Background:**

Recent trends in the incidence of lung cancer have been reported despite the decreasing rate of smoking. Lung cancer is ranked among the top causes of cancer-related deaths. The ratio of adenocarcinoma to squamous cell carcinoma, as well as the ratio of women to men, is still increasing. Human papillomavirus (HPV) has been discovered in lung cancer tissues and blood specimens, particularly in Eastern countries. However, the association between HPV infection and lung adenocarcinoma remains unclear.

**Methods:**

This population-based cohort study was conducted using data from Taiwan's single-payer national health insurance and cancer registry databases. Data on HPV infection, cancer, sex, age, comorbidities, urbanization, and occupation were collected. The cumulative incidence rates were generated using Kaplan–Meier curves and log-rank tests. COX regression analysis was used to estimate the hazard ratios of factors associated with cancer occurrence. We used data from 2007 and 2015. The cases were matched with sex and age in a 1:2 manner with 939,874 HPV+ and 1,879,748 HPV– individuals, respectively.

**Results:**

The adjusted hazard ratios [95% confidence interval (CI)] for HPV infection in all lung cancers were 1.539 (1.436–1.649), male lung cancer 1.434 (1.312–1.566), female lung cancer 1.742 (1.557–1.948), squamous cell carcinoma (SCC) 1.092 (0.903–1.320), male SCC 1.092 (0.903–1.320), female SCC 0.949 (0.773–1.164), adenocarcinoma 1.714 (1.572–1.870), male adenocarcinoma 1.646 (1.458–1.858), and female adenocarcinoma 1.646 (1.458–1.858). The highest adjusted hazard ratio for lung cancer was chronic obstructive pulmonary disease (COPD) 1.799 (1.613–2.007), followed by male sex 1.567 (1.451–6.863) and HPV infection. The highest adjusted hazard ratio for adenocarcinoma was HPV infection 1.714 (1.572–1.870), followed by COPD 1.300 (1.102–1.533), and for SCC, male sex 5.645 (4.43–3.37), followed by COPD 2.528 (2.002–3.192).

**Conclusion:**

Our study showed that HPV infection was associated with the occurrence of adenocarcinoma of the lung in both men and women but was not associated with SCC of the lung.

## Introduction

Cancer is one of the primary causes of death (1) and its etiology involves multiple factors. Prominent factors include infectious agents, such as tuberculosis, human immunodeficiency virus, Epstein-Barr virus, hepatitis viruses, *Helicobacter pylori*, and human papillomavirus (HPV), which contribute to 20–25% of all globally registered cancer cases (2). Viruses are responsible for 12% of all human cancers, and HPV is responsible for approximately 30% of all cancers related to infectious agents (2). In the United States, 14.1 million new incidences of HPV infections were reported in 2008 and increased to 24.4 million by 2018 (3, 4). HPV accounts for the majority of sexually transmitted disease cases in the United States (3). In summary, approximately 79 million women between the ages of 14 and 59 years contracted HPV, with the highest prevalence being in women between the ages of 20 and 24 years (5). HPV infection has been reported to correlate with head and neck, esophageal, and anogenital cancer (6–10).

Among all cancers, lung cancer is the most lethal in Taiwan (1). In addition to smoking, passive smoking, indoor fume pollution, and genetics, HPV has been proposed to be one of the etiologies of lung cancer (11–13). HPV was detected in lung squamous cell carcinoma in the pathological form of condylomas in 1979 (14). The meta-analyses conducted by Tsyganov et al. and Karnosky et al. found a correlation between HPV and lung cancer, as people with HPV had 5.4 and 4.7 times greater likelihood of developing lung cancer compared to people without. In addition, detection variation was shown based on geographic areas and HPV subtypes, with people living in Asia having a greater likelihood of having HPV. Currently, studies on the molecular mechanisms underlying HPV-related lung cancer are in progress (15, 16). However, to date, no large-scale population-based studies have been conducted to demonstrate the association between HPV and various histological subtypes of lung cancer.

Although an association between HPV infection and the occurrence of Taiwanese lung cancer has been reported in a small number of clinical tumor tissues, no epidemiological data has been found to support the findings (17–19). Previously, the 2001–2004 Nationwide Population-Based Cohort Study showed a correlation between HPV and lung cancer in female individuals, whereas a correlation between lung cancer and chronic obstructive pulmonary disease (COPD) was found in male individuals (20). Furthermore, data from the National Cancer Registry Database, which includes cancer subtypes, support the association between HPV and lung cancer in cancer diagnosis. Additionally, lung adenocarcinoma was found to differ from squamous cell carcinoma (SCC) in terms of etiology, distribution, genetics, therapeutic medications, and survival (21). In this study, we investigated whether there was a relationship between HPV-related lung cancer and sex and lung cancer histological type.

## Materials and Methods

### Data Source

This study analyzed datasets managed by the Health and Welfare Sciences Center, Ministry of Health, Taiwan. Datasets included 2006–2015 ambulatory care expenditures by visits, inpatient expenditures by admissions, details of ambulatory care orders, details of inpatient orders, registry for beneficiaries, cause of death data, and Taiwan Cancer Registry—Long/Short Form. Each patient was identified with a Taiwanese national identification number that was coded to an encrypted identification number to protect personal data. The first database was the 2007–2015 National Health Insurance Research Dataset, which included 26 million unique identification numbers managed by the Health and Welfare Sciences Center. The second database was the 1979–2015 Taiwan Cancer Registry managed by the Health Promotion Administration, Ministry of Health and Welfare, Taiwan.

### ICD-9 International Classification of Diseases, Ninth Revision

Human papillomavirus (ICD-9-CM: 079.4, 078.1, 795.05, 795.09, 795.15, 795.19, 796.75, and 796.79), lung cancer (ICD-9: 162), cervical cancer (ICD-9: 180. X), and head and neck cancer (ICD-9: 195. X) were chosen as the standards since HPV infection is known to induce cervical and head and neck cancers (4). Comorbidities included chronic obstructive pulmonary disease (COPD; ICD-9: 492–496), hyperlipidemia (ICD-9: 272. X), ischemic heart disease (IHD; ICD-9: 411, 413–4), hypertension (ICD-9: 401), diabetes mellitus (DM; ICD-9: 250), ischemic stroke (ICD-9: 433–4, 436), liver disease (ICD-9: 571), peptic ulcer (ICD-9: 533.), gastrointestinal (GI) hemorrhage (ICD-9: 578. X), gout (274. X), and renal disease (ICD-9: 580–589).

Stratifications only included cancer subtypes of squamous cell carcinoma and adenocarcinoma. Other subtypes, such as non-small cell carcinoma type unknown, large cell, and small cell carcinoma, were excluded because the disease incidence numbers were not large enough to be included in the statistics.

### Study Design and Ethical Considerations

For the population-based cohort study design, the index date was identified as the starting point, and the incidence of cancer was from the index date to 31 December 2015. The male population was also excluded from the cervical cancer statistics. The study protocol was reviewed and approved by the institutional review board of Chung Shan University Hospital (IRB CS13168). All patient data from the National Health Insurance Research Dataset were de-identified; therefore, the requirement for signed informed consent was waived.

### Statistical Analysis

SAS 9.4 (SAS Institute Inc., Cary, North Carolina) was used for statistical analyses in this study. For the design of the cohort study, the index date was identified as the starting point and the incidences of cancer from the index date to 31 December 2015 were included. Patients with HPV before 2008 or those with cancer before the diagnosis of HPV were excluded. The non-infected cases were matched with HPV-infected cases according to age, sex, and index date with structured query language (SQL) in a 2:1 manner. Patients with cancer onset before the index date were excluded. In this study, comparisons were made between HPV-infected and non-HPV-infected individuals. Demographic data were compared using the chi-square test for categorical data and Student's *t*-test for numerical data. The relative risk of cancer was calculated using a multiple Cox regression model. The cumulative incidence rates of cancer were generated using Kaplan–Meier curves and tested by the long-tank test.

## Results

**Figure 1:**
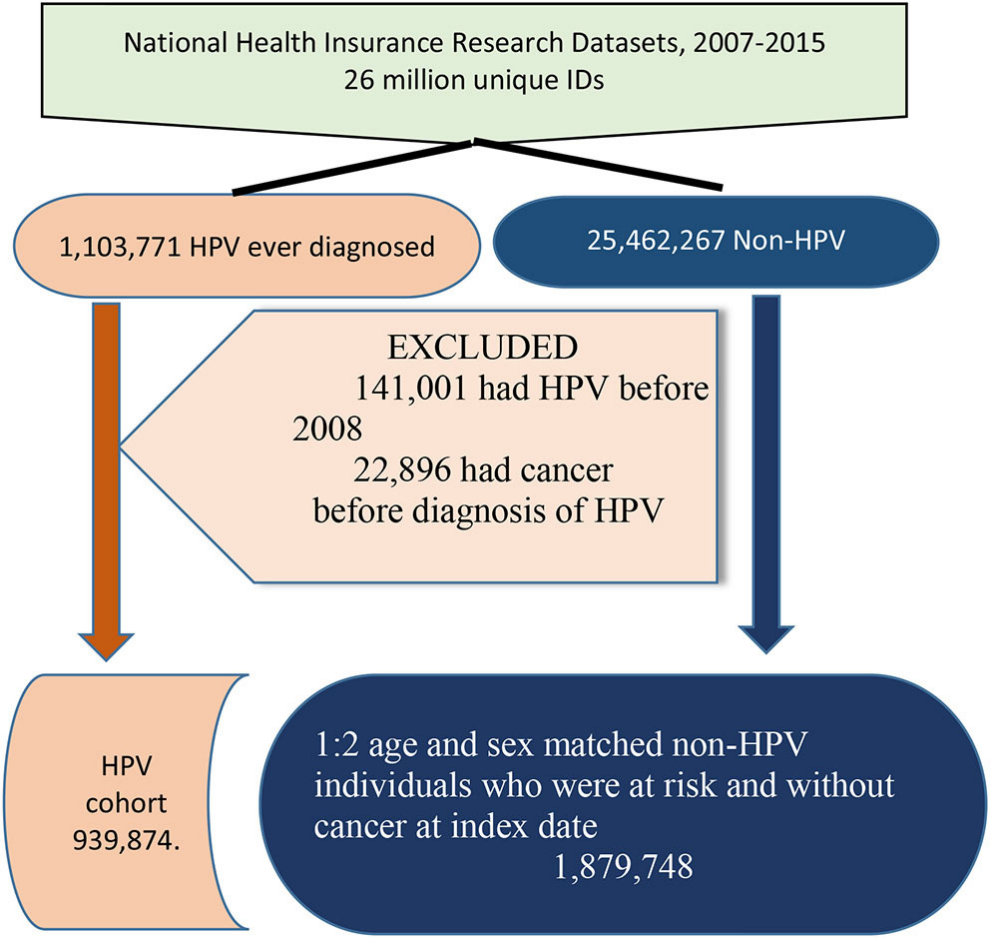
The algorithm of enrollment.

During the period of 2007–2017, 1,103,771 people tested positive for HPV, yielding an incidence rate of 4.2% (Figure 1). Among those with HPV, 141,001 individuals who contracted HPV before 2008 were excluded from the study. Similarly, 22,896 individuals diagnosed with lung cancer before HPV infection were excluded from this study. Subsequently, the remaining 939,873 individuals with HPV were included in the study cohort, and we included double the number of individuals without HPV who were matched on sex, age, and index date (control group). However, identifying HPV infection was challenging because most HPV types that developed viral warts were coded 078.10 and 078.19, and the latter accounted for 91.04% (Table 1). The age and sex of HPV-positive and HPV-negative individuals were perfectly matched (*p* = 1.0,000) (Table 2). A positive correlation was found between the degree of urbanization and incidence rate (*p* < 0.0001). Officers and laborers had more HPV infections than farmers or unemployed workers did (*p* < 0.0001). As for comorbidities, the incidence of HPV infection was positively correlated with hyperlipidemia, ischemic heart disease, hypertension, peptic ulcer, gastrointestinal bleeding, chronic kidney disease, and COPD but not with diabetes mellitus.

**Table 1:** HPV ICD-9 Code, 2007–2015.

**ICD-9**	**Frequency**	**Person (*N*)**	**aHR (95% CI) for cancer**
078.10 viral wart, unspecified	9,252,831 (75.46%)	903,660	1.579(1.544–1.616)
078.19 other specified viral warts	1,911,742 (15.59%)	226,622	1.348(1.298–1.401)
078.1X viral warts	677,015 (5.52%)	83,517	1.365(1.296–1.437)
078.11 condyloma acuminatum	411,947 (3.36%)	51,575	1.739(1.618–1.869)
079.4X HPV in conditions classified elsewhere and of unspecified site	8,246 (0.07%)	1,090	3.057(2.172–4.301)

*One person could have multiple codes. The total number of persons > recruited. ICD-9 795.X and 796.X were rarely used in Taiwan*.

**Table 2:** Demography of people with and without HPV.

		**HPV**	**Non-HPV**	***P***
Total		939,874	1,878,748	
**Gender**				
	Male	472,420(50.26%)	944,840(50.26%)	
	Female	467,454(49.74%)	934,908(49.74%)	
**Age**				
	<20	256,500(27.29%)	513,000(27.29%)	1.0,000
	20–40	334,686(35.61%)	669,372(35.61%)	
	40–60	234,575(24.96%)	469,150(24.96%)	
	60–80	95,755(10.19%)	191,510(10.19%)	
	≥80	18,358(1.95%)	36,716(1.95%)	
**Co–morbidities**				
	Ischemic heart disease	39,334(4.19%)	63,872(3.40%)	<0.0,001
	Hypertension	112,709(11.99%)	205,015(10.91%)	<0.0,001
	Hyperlipidemia	83,114(8.84%)	133,790(7.12%)	<0.0,001
	Stroke	20,246(2.15%)	40,087(2.13%)	0.2,386
	Diabetes mellitus	46,782(4.98%)	96,584(5.14%)	<0.0,001
	Abnormal liver function	45,661(4.86%)	91,126(4.85%)	0.7,009
	Peptic ulcer	74,302(7.91%)	122,250(6.50%)	<0.0,001
	GI bleeding	5,723(0.61%)	10,826(0.58%)	0.0,006
	Renal failure	8,191(0.87%)	15,200(0.81%)	<0.0,001
	Chronic kidney diseases	14,747(1.57%)	25,740(1.37%)	<0.0,001
	Gout	28,685(3.05%)	49,184(2.62%)	<0.0,001
	COPD	18„607(1.98%)	32,326(1.72%)	<0.0,001
**Urbanization**				
	Urban 1	319,694(34.01%)	542,433(28.86%)	<0.0,001
	2	290,511(30.91%)	587,017(31.23%)	
	3	162,859(17.33%)	340,505(18.11%)	
	4	109,824(11.68%)	246,844(13.13%)	
	5	13,197(1.40%)	36,687(1.95%)	
	6	23,620(2.51%)	70,850(3.77%)	
	Rural 7	20,169(2.15%)	55,412(2.95%)	
**Occupation**				
	Officer	79,002(8.41%)	111,753(5.95%)	<0.0,001
	Laborer	618,860(65.84%)	1,183,084(62.94%)	
	Farmer	94,224(10.03%)	237,256(12.62%)	
	Low income	7,928(0.84%)	20,772(1.11%)	
	Unemployed	122,182(13.00%)	29,4431(15.66%)	
	Others	17,678(1.88%)	32,452(1.73%)	

**Figure 2:**
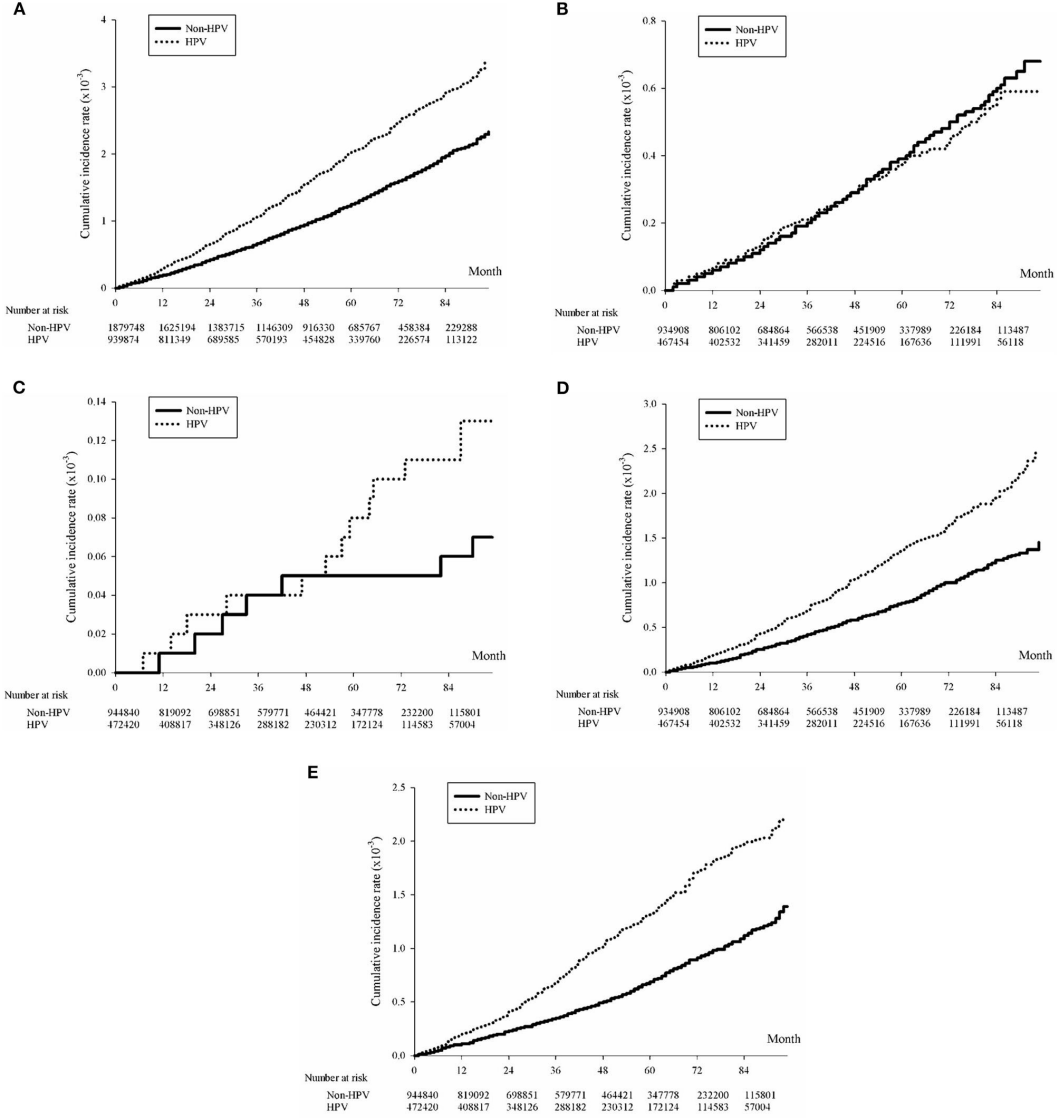
**(A)** Kaplan-Meier curve of lung cancer cumulative incidence rates with and without HPV infection. Lung Cancer, *p* < 0.0001; **(B)** Kaplan-Meier curve of male squamous cell carcinoma cumulative incidence rates with and without HPV infection. Squamous Cell Carcinoma, male, *p* = 0.6886; **(C)** Kaplan-Meier curve of female squamous cell carcinoma cumulative incidence rates with and without HPV infection. Squamous Cell Carcinoma, female, *p* = 0.0643; **(D)** Kaplan-Meier curve of male adenocarcinoma cumulative incidence rates with and without HPV infection. Adenocarcinoma, male, *p* < 0.0001 and **(E)** Kaplan-Meier curve of female adenocarcinoma cumulative incidence rates with and without HPV infection. Adenocarcinoma, female, *p* < 0.0001.

Human papillomavirus was associated with an increased risk of lung, cervical, and head and neck cancer (Table 3). The hazard ratio (HR) of cervical cancer between individuals with and without HPV was 2.225 (2.008–2.464). The incidence rate of cervical cancer was 41.65/100,000 person-years for individuals with HPV compared to 18.89/100,000 person-years for individuals without HPV. Similarly, the HR of head and neck cancer was 1.527 (1.436, 1.649, *p* < 0.01), with an incidence rate of 21.86/100,000 person-years in the HPV positive individuals and an incidence rate of 14.32/100,000 person-years in HPV-negative individuals. The HR for indiscriminate lung cancer was 1.539 (1.436–1.649, *p* < 0.001), with an incidence rate of 39.44/100,000 person-years for individuals with HPV and an incidence rate of 25.54/100,000 person-years for individuals without HPV. Among all lung cancer individuals, men had an HR of 1.539 (1.439–1.649, *p* < 0.001) between HPV-positive and HPV-negative individuals, with an incidence rate of 47.47/100,000 person-years for HPV-positive individuals and an incidence rate of 33.40/100,000 person-years for HPV-negative individuals. In comparison, women had an HR of 1.742 (1.557–1.948), with an incidence of 31.57/100,000 person-years for HPV-positive individuals and an incidence rate of 17.83/100,000 person-years for HPV-negative individuals. The incidence rate was higher in HPV-positive than in HPV-negative cervical cancer (*p* < 0.001) (Supplementary Figure 1), head and neck cancer (Supplementary Figure 2), and lung cancer (Figure 2A).

**Table 3:** Risk of cancer occurred for people with and without HPV infection.

	**HPV**				**Non–HPV**					
	**PM**	**Event**	**Incidence rate^†^**		**PM**	**Event**	**Incidence rate^†^**		**Crude HR**	**Adjusted HR**
**Head and Neck Cancer**	44,357,317	808	21.86 (20.40–23.42)		89,136,676	1,064	14.32 (13.49–15.21)		1.527(1.393–1.673)^*^	1.595(1.453–1.749)^*^
**Cervical Cancer**	22,388,511	777	41.65 (38.82–44.68)		45,020,697	712	18.98 (17.63–20.42)		2.195(1.983–2.430)^*^	2.225(2.008–2.464)^*^
**Lung Cancer–All**	44,357,317	1,458	39.44 (37.47–41.52)		89,136,676	1,897	25.54 (24.41–26.71)		1.546(1.444–1.655)^*^	1.539(1.436–1.649)^*^
Male	21,968,806	869	47.47(44.41–50.73)		44,115,979	1,228	33.40(31.59–35.32)		1.422(1.304–1.551)^*^	1.434(1.312–1.566)^*^
Female	22,388,511	589	31.57(29.12–34.23)		45,020,697	669	17.83(16.53–19.24)		1.772(1.586–1.980)^*^	1.742(1.557–1.948)^*^
Age <60 years	39,453,993	519	15.79(14.48–17.20)		79,157,462	611	9.26(8.56–10.03)		1.705(1.517–1.917)^*^	1.664(1.478–1.873)^*^
Age ≥60 years	4,903,324	939	229.81(215.57–244.98)		9,979,214	1,286	154.64(146.42–163.33)		1.491(1.371–1.622)^*^	1.483(1.361–1.616)^*^
**Squamous Cell Carcinoma**	44,357,317	167	4.52(3.88–5.26)		89,136,676	326	4.39(3.94–4.89)		1.030(0.855–1.241)	1.092(0.903–1.320)
Male	21,968,806	138	7.54 (6.38–8.91)		44,115,979	289	7.86 (7.01–8.82)		0.960(0.784–1.175)	0.949(0.773–1.164)
Female	22,388,511	29	1.55 (1.08–2.24)		45,020,697	37	0.99 (0.71–1.36)		1.576(0.969–2.563)	1.540(0.945–2.509)
Age <60 years	39,453,993	36	1.09 (0.79–1.52)		79,157,462	62	0.94 (0.73–1.21)		1.165(0.773–1.757)	1.151(0.762–1.739)
Age ≥60 years	4,903,324	131	32.06 (27.01–38.05)		9,979,214	264	31.74 (28.14–35.82)		1.013(0.822–1.249)	0.984(0.796–1.215)
**Adenocarcinoma**	44,357,317	981	26.54(24.93–28.25)		89,136,676	1,119	15.06(14.21–15.97)		1.763(1.618–1.921)^*^	1.714(1.572–1.870)^*^
Male	21,968,806	489	26.71 (24.44–29.19)		44,115,979	581	15.80 (14.57–17.14)		1.691(1.500–1.908)^*^	1.646(1.458–1.858)^*^
Female	22,388,511	492	26.37 (24.14–28.81)		45,020,697	538	14.34 (13.18–15.60)		1.841(1.629–2.080)^*^	1.812(1.603–2.049)^*^
Age <60 years	39,453,993	415	12.62 (11.46–13.90)		79,157,462	441	6.69 (6.09–7.34)		1.889(1.652–2.160)^*^	1.847(1.614–2.112)^*^
Age ≥60 years	4,903,324	566	138.52 (127.57–150.42)		9,979,214	678	81.53 (75.62–87.90)		1.705(1.525–1.907)^*^	1.653(1.477–1.850)^*^

*PM, person–months*.

* *statistic significance with p < 0.05.*

† *crude incidence rate, per 100,000 person–years*.

The results of subgroup analysis indicated that the incidence rate of adenocarcinoma was 3.43 times higher than that of squamous cell carcinoma in the HPV-negative population and 5.87 times higher in the HPV-positive population (Table 3). For squamous cell carcinoma, the incidence rate was lower in the HPV-positive population (7.54/100,000 person-years) than in the HPV-negative population (7.86/100,000 person-years) among men (Table 3). In comparison, the incidence rate was higher in the HPV-positive population (1.55/100,000 person-years) than in the HPV-negative population (0.99/100,000 person-years) among women. The HR of squamous cell carcinoma of the lung was 0.949 (0.773, 1.164, *p* = 0.6,886) in the male population and 1.540 (0.945–2.509, *p* = 0.0,643) in the female population, which was almost significant (Figures 2B,C). Next, the HPV incidence rate of squamous cell carcinoma was stratified by age ≥ 60 years. The incidence rate in the HPV-positive population (1.09/100,000 person-years) was higher than that in the HPV-negative population (0.94/100,000 person-years) for the group aged < 60 years. A significant outcome was found among the group above the age of 60 years, comparing the incidence rate of the HPV-positive group (32.06/100,000 person-years) with the HPV-negative group (31.74/100,000 person-years). The HR for squamous cell carcinoma was 1.151 (0.762–1.739) for the population below the age of 60 and 0.984 (0.796–1.215) years for the population above the age of 60 years.

The incidence of adenocarcinoma was also compared between HPV-positive and HPV-negative individuals. Stratified by sex, the incidence rate in the HPV-positive population (26.71/100,000 person-years) was higher than that in the HPV-negative population (15.80/100,000 person-years) among both men and women [HPV (+): 26.37/100,000 person-years and HPV (–): 14.34/100,000 person-years] (Table 3). In addition, higher incidence rates of adenocarcinoma than squamous cell carcinoma were observed in HPV-positive individuals of both sexes. The HR of adenocarcinoma was 1.646 (1.458–1.858, *p* < 0.0,001) in the male population and 1.812 (1.603–2.049, *p* < 0.0,001) in the female population (Figures 2D,E). Stratified by age above and below the age of 60 years, the incidence rate of adenocarcinoma was higher in the HPV-positive population than in the HPV-negative population for both ages above and below, with the group above the age of 60 years [HPV (+): 138.52/100,000 person-years and HPV (–): 81.53/100,000 person-years] being more significant than below [HPV (+): 12.62/100,000 person-years and HPV(–): 6.69/100,000 person-years]. The HR of adenocarcinoma was 1.847 (1.614–2.112) in the age group below 60 years and 1.653 (1.477–1.850) in the age group above 60 years.

The factors associated with lung cancer were determined by COX regression analysis, demonstrated with the adjusted hazard ratio (aHR), 95% confidence interval (CI), and *p*-value (*p*). The contributing factors of lung cancer included HPV infection, sex, age, occupation, and other comorbidities (Table 4). Individuals with HPV had a higher risk of lung cancer compared to individuals without HPV (aHR = 1.539, 1.436–1.649), *p* < 0.0,001). Similarly, male individuals had a higher risk of lung cancer compared to their female counterparts (aHR = 1.567, 1.459–1.683, p < 0.0,001). The aHR of lung cancer was also proportional to the stratified age. With age 20–40 years as a reference, an aHR of 0.046 (0.017–0.124, *p* < 0.0,001) was observed among individuals <20 years of age, an aHR of 12.679 (10.415–15.434, *p* < 0.0,001) was observed among individuals aged 40 and 60 years, an aHR of 45.477 (37.294–55.457, *p* < 0.001) was observed among individuals between the ages of 60 and 80 years, and an aHR of 73.521 (59.192–91.319, *p* < 0.0,001) was observed among individuals above the age of 80 years. Furthermore, individuals covered by the insurance of the farmers' and fishermen's unions had greater risk (aHR = 1.242, 1.111–1.388, *p* = 0.0,001) compared to individuals with laborers' union insurance. Among the common comorbidities listed in Table 4, individuals with hypertension (aHR = 1.183, 1.090–1.285, *p* < 0.0,001), COPD (aHR = 1.799, 1.613–2.007, *p* < 0.0,001), and peptic ulcer (aHR = 1.178, 1.076–1.290, *p* = 0.0,004) were at a greater risk of lung cancer than those without the respective comorbidities. However, the results were dissimilar between cancer cell types.

**Table 4:** Adjusted hazard ratio for lung cancer.

			**Lung cancer, all**				**Squamous cell carcinoma**				**Adenocarcinoma**		
			**aHR**	**95% CI**	**p**		**aHR**	**95% CI**	**p**		**aHR**	**95% CI**	**p**
**HPV**	with vs. without		1.539	1.436–1.649	<0.0001^*^		1.092	0.903–1.320	0.3,653		1.714	1.572–1.870	<0.0,001^*^
**Gender**	Male vs. Female		1.567	1.459–1.683	<0.0,001^*^		5.645	4.343–7.337	<0.0,001^*^		1.025	0.939–1.119	0.5798
**Age**													
	<20		0.046	0.017–0.124	<0.0,001^*^		0.239	0.028–2.045	0.1,913		0.016	0.002–0.115	<0.0,001^*^
	20–40		Reference				Reference				Reference		
	40–60		12.679	10.415–15.434	<0.0,001^*^		26.152	10.621–64.394	<0.0,001^*^		12.803	10.186–16.092	<0.0,001^*^
	60–80		45.477	37.294–55.457	<0.0,001^*^		155.166	63.540–378.919	<0.0,001^*^		37.078	29.336–46.864	<0.0,001^*^
	≥80		73.521	59.192–91.319	<0.0,001^*^		267.473	107.162–667.604	<0.0001^*^		51.748	39.676–67.494	<0.0,001^*^
**Urbanization**													
Urban	1		Reference				Reference				Reference		
	2		0.928	0.843–1.023	0.1,321		1.282	0.971–1.692	0.0,798		0.813	0.722–0.917	0.0,007^*^
	3		0.948	0.843–1.067	0.378		1.579	1.153–2.162	0.0,044^*^		0.844	0.729–0.977	0.0,231^*^
	4		0.914	0.802–1.041	0.1,759		1.135	0.797–1.616	0.4,842		0.746	0.631–0.883	0.00,068^*^
	5		1.007	0.800–1.267	0.9,547		1.36	0.792–2.336	0.2,648		0.915	0.677–1.236	0.5,616
	6		0.84	0.688–1.025	0.0,856		1.253	0.788–1.993	0.3,409		0.683	0.522–0.894	0.0,056
Rural	7		1.093	0.889–1.342	0.3,996		1.792	1.116–2.877	0.0,158^*^		0.813	0.612–1.080	0.1,529
**Occupation**													
	Officer		0.978	0.848–1.128	0.7,635		0.594	0.356–0.994	0.0,473^*^		1.098	0.929–1.298	0.2725
	Laborer		Reference				Reference				Reference		
	Farmer		1.242	1.111–1.388	0.0,001^*^		1.692	1.290–2.220	0.0,001^*^		1.182	1.021–1.368	0.0255^*^
	Low income		1.191	0.807–1.758	0.3,794		2.682	1.361–5.283	0.0,043^*^		0.794	0.425–1.482	0.469
	Unemployed		1.049	0.956–1.151	0.3,116		1.082	0.842–1.389	0.5,393		1.089	0.970–1.223	0.1,482
	Others		1.223	0.927–1.614	0.1,543		0.959	0.393–2.339	0.9,259		1.327	0.956–1.844	0.0,912
**Co–morbidity**													
	Ischemic heart disease		1.015	0.919–1.121	0.7,692		0.889	0.692–1.141	0.3,543		1.097	0.963–1.249	0.1,622
	Hypertension		1.183	1.090–1.285	<0.0,001^*^		1.012	0.823–1.245	0.9,083		1.242	1.117–1.380	<0.0,001^*^
	Hyperlipidemia		0.979	0.894–1.073	0.6,496		1.003	0.794–1.267	0.9,799		1.052	0.937–1.181	0.39
	Stroke		1.105	0.984–1.241	0.0,914		1.167	0.891–1.530	0.2,619		1.085	0.926–1.271	0.3117
	Diabetes mellitus		1.072	0.974–1.180	0.1,553		1.25	0.992–1.577	0.059		0.976	0.860–1.109	0.7,113
	Abnormal liver function		1.103	0.985–1.236	0.0,901		1.25	0.948–1.649	0.1,137		1.159	1.004–1.338	0.0,444
	Peptic ulcer		1.178	1.076–1.290	0.0,004^*^		1.03	0.814–1.303	0.8,069		1.222	1.088–1.372	0.0,007^*^
	GI bleeding		0.81	0.597–1.099	0.176		0.395	0.146–1.066	0.0,666		0.838	0.553–1.271	0.4,063
	Renal failure		1.036	0.758–1.417	0.8,233		0.893	0.410–1.946	0.7,764		0.913	0.588–1.418	0.6,867
	Chronic kidney diseases		0.988	0.770–1.267	0.9,217		0.873	0.476–1.602	0.6,616		0.975	0.695–1.368	0.883
	Gout		0.992	0.874–1.126	0.8,964		1.19	0.900–1.572	0.2,217		0.918	0.769–1.096	0.3,425
	COPD		1.799	1.613–2.007	<0.0,001^*^		2.528	2.002–3.192	<0.0,001^*^		1.300	1.102–1.533	0.0,018^*^

*COX Regression*.

* *p < 0.05*.

In individuals with squamous cell carcinoma, sex, age, urbanization, insurance coverage, and comorbidities were contributing factors (Table 4). Male individuals had an aHR of 5.645 (4.343–7.337, *p* < 0.001) compared to their female counterparts. An aHR was also proportional to age. With age 20–40 as a reference, an aHR of 26.152 (10.621–64.394, *p* < 0.0,001) was observed between the ages of 40 and 60 years, an aHR of 155.166 (63.540–378.919, *p* < 0.0,001) between the ages of 60 and 80 years, and an aHR of 267.473 (107.162–667.473, *p* < 0.0,001) above the age of 80 years. The degree of urbanization, on a scale of 1–7 was also a contributing factor. With 1 as a reference, the degree of urbanization of 3 had an aHR of 1.579 (1.153–2.162, *p* = 0.0,044) and 7 had an aHR of 1.792 (1.116–2.877, p < 0.0,158). Additionally, aHR varied across insurance plans. Compared to the insurance covered by the Laborers' Union, the insurance covered by the government (aHR = 0.594, 0.356–0.994, *p* = 0.0,437) showed a significantly higher aHR, farmers' union (aHR = 1.692, 1.290–2.220, *p* = 0.0,001), and low-income household subsidies (aHR = 2.682, 1.361–5.283, *p* = 0.0,043). Among the common comorbidities listed, COPD had an aHR of 2.528 (2.002–3.192, *p* < 0.0,001) with the highest aHR among all comorbidities.

Significant causes of adenocarcinoma included HPV infection, age, urbanization, insurance coverage, and comorbidities (Table 4). HPV-infected adenocarcinoma individuals had an aHR of 1.714 (1.572–1.870, *p* < 0.0,001) compared to non-HPV individuals, which was the highest among all factors, except age. An aHR was also proportional to age. With age 20–40 years as a reference, an aHR of 0.016 was seen in individuals below the age of 20, an aHR of 26.12.803 (10.186–16.092, *p* < 0.0,001) between the ages of 40 and 60 years, an aHR of 37.078 (29.336–46.864, *p* < 0.0,001) between the ages of 60 and 80 years, and an aHR of 51.748 (39.676–67.494, *p* < 0.0,001) above the age of 80 years. The degree of urbanization also varied with the survival rate of individuals with adenocarcinoma. On a scale of 1–7, degree 2 (aHR = 0.813, 0.722–0.917, *p* = 0.0,007), degree 3 (aHR = 0.844, 0.729–0,944, *p* = 0.0231), and degree 4 (aHR = 0.746, 0.631–0.883, *p* = 0.0,006) had significantly lower aHR. Furthermore, individuals covered by the insurance of the Farmers' Union had an aHR of 1.182 (1.021–1.369, *p* = 0.0,255) compared to Laborers' Union. Among the common comorbidities, hypertension (aHR = 1.242, 1.117–1.380, *p* < 0.0,001), abnormal liver function (aHR = 1.159, 1.004–1.338, *p* = 0.0,444), COPD (aHR = 1.300, 1.102–1.533, *p* = 0.0,018), and peptic ulcer (aHR = 1.222, 1.088–1.372, *p* = 0.0,007) all exhibited higher rates.

The contributing factors analyzed in indiscriminate lung cancer, squamous cell carcinoma, and adenocarcinoma individuals were similar to those of cervical cancer, including HPV infection, age, insurance coverage, and comorbidities (Supplementary Table S1). The aHR of HPV-positive cervical cancer individuals was 2.225 (2.008–2.464, *p* < 0.0,001) with HPV-negative counterparts as a reference. Compared to the age of 20–40 years, however, the maximum aHR was found at the age of 40–60 years (aHR = 1.124, 1.001–1.262, *p* = 0.0,481), unlike the case in all lung cancer. In terms of insurance coverage, except for those covered by the government that had a lower aHR of 0.720 (0.565–0.917, *p* = 0.0,077), cervical cancer individuals covered by all other insurance plans had a relatively higher aHR. Furthermore, renal failure was the main contributor to comorbidities, with an aHR of 2.725 (1.235–6.009, *p* = 0.0,130).

## Discussion

In this study, we utilized a nation-based cohort study to investigate the important etiological role of HPV in lung adenocarcinoma. Our study found that HPV was associated with adenocarcinoma of the lung in both sexes in Taiwan, with a stronger association in the female population, whereas SCC occurred more frequently in men with COPD.

Smoking is a well-established leading cause of lung cancer. However, approximately 50% of the global male population and 10% of the global female population are reported to possess smoking behaviors (22). The smoking rate in the Asian female population was 2%. The smoking rate in Taiwan has been surveyed since 1990 with a continual downward trend. According to data from 1990 to 2018, the smoking rate in Taiwan decreased from 59.4% to 23.4% in men and 3.8% to 2.4% in women (22). The leading cause of death before 2011 in the male population was hepatoma, followed by lung cancer, and lung cancer was the leading cause of death after 2011 (1). The standard lung cancer incidence rates were 44.2/10^6^ people in 2011 and 43.5/10^6^ people in 2014. In contrast, the standard female death rate increased and stabilized, with 12.0/10^6^ people in 1981, 17.3/10^6^ people in 1996, 17.5/10^6^ people in 2001, 16.5/10^6^ people in 2011, and 15.3/10^6^ people in 2019. Before 1986, cervical cancer was the leading cause of death in the female population, followed by lung cancer, and lung cancer became the leading cause of death after 1986. The standard incidence rate of lung cancer in women was 24.8/10^6^ people in 2011 and 28.3/10^6^ people in 2014 (1, 22).

According to a study from the Taiwan Cancer Registry from 1996 to 2008, the male-to-female ratio of the lung cancer incidence rate decreased due to the progressive discussion surrounding sex issues. However, the annual incidence rate of adenocarcinoma has increased in both sexes, with a greater increase in the female population. In contrast, the annual incidence rate of squamous cell carcinoma decreases in the female population and remains constant in the male population (21). Despite the drop in the smoking rate, the incidence rate of lung cancer increased from 45.04/10^6^ people in 2002 to 49.86/10^6^ people in 2011 (22). Notably, the incidence rate increased more rapidly in the female population than in the male population, with an annual 0.2% change in the male population and a 2.0% change in the female population. Overall, the incidence of adenocarcinoma increased from 43.45% to 64.89% between 2002 and 2014 (22).

Our results were temporally different from those of previous studies, in which our study included data from 2006 to 2015 compared to previous studies from 2001 to 2004 (20). During this period, the incidence rate of lung cancer and death rate continued to increase despite the decrease in the smoking rate, suggesting an increase in the incidence rate of non-smokers. The incidence rate of lung cancer in men was lower in this study than in a previous study (20). The latter also supports an association between lung cancer and HPV. Nonetheless, this study indicated an increase in the lung cancer rate among the non-smoking (COPD) population for both men and women, with women having a more rapid increase than men. In terms of cancer cell type, the incidence rate of squamous cell carcinoma decreased in this study, but the incidence rates of adenocarcinoma and lung cancer in the non-smoking population increased, suggesting an association between HPV infection and lung adenocarcinoma in the non-smoking population. Contrary to a previous study, HPV infection was also associated with adenocarcinoma in the male population, which was supported by an increase in the incidence rate of adenocarcinoma in the non-smoking male population.

Human papillomavirus is prevalent worldwide, accounting for approximately 10% of the global population (23). There are 170 documented types of HPV (7) with additional subtypes. HPV types in most oncologic cell studies, mainly from investigating cervical cancer instead of lung cancer, include 12 viral types closely related to malignant neoplasms (HPV-16, 18, 31, 33, 35, 39, 45, 51, 52, 58, and 59). In particular, HPV-16 and 18 are associated with a high risk of developing 63% of all cancers. HPV-16 is the most extensively studied, whereas types 31, 33, 45, 52, and 58 contribute to the development of 10% of all cancers. The most prevalent HPV types are low-risk (LR)-HPVs-6 and 11, which cause 90% of genital and oral pharyngeal papillomas (6). In most of the studies, samples are collected from semen or vaginal swabs. In our studies, *verruca vulgaris* 1, 2, 4, 28, and 30, which resulted in common wart formation, were the most frequently investigated types of viruses. The treatment of such viral strains is estimated to cost 1.7 billion a year in the United States. Among the 86 tissue studies of lung cancer and high-risk HPV, including types 16, 18, 31, and 33 and types 16 and 18, were present in 58 articles (15). However, when HPV-6 was detected, HPV-16 and 18 were also detected in 74% (66/89) of the lung cancer tissues. Similarly, they were detected in 49% (21/43) of the lung cancer tissues when HPV-11 was detected. Some negative testing results may be due to the fewer subtypes tested in the studies. However, HPV-6 and 11 are predominant in lung adenocarcinoma; however, subtype and 1, 2, 4, 28, and 30 have rarely been studied in lung cancer tissue.

The prevalence of HPV has also been suggested to be associated with the prevalence of cervical cancer. The prevalence rates of HPV differ across continents, with Asia having the highest prevalence rate, followed by Latin America, Australia, Europe, and North America (15, 16). The incidence of lung cancer varies geographically. EGFR-mutant lung cancer, for instance, is more prevalent in Asia than in North America. Hence, the study of HPV-contracted lung cancer may be more important in areas with dense populations. This also implies that adenocarcinomas in Taiwan may be associated with HPV infection.

Interestingly, the localization of the virus does not fixate on the epithelium. It may travel beyond superficial layers to the semen, amniotic fluid, placenta, umbilical cord, fetus, breast milk, and blood. Evidence from about 10 studies revealed multiple types of HPV, including 6, 11, 16, 18, and other 10s in blood monocytes and circulatory tumor cells of normal, cervical, and lung cancer subjects (2, 24). HPV invades through epidermal defects or potentially through blood circulation and anchors to the basal membrane. However, asymptomatic HPV due to insufficient HPV concentration may escape clinical detection and lead to the development of cancer.

We suggest that squamous cell carcinoma is infected *via* direct contact with HPV, whereas adenocarcinoma develops through transmission in the bloodstream. Squamous cell carcinoma differs from adenocarcinoma in various aspects, yet HPV has been detected in equal amount in tissue studies: 25.8% of squamous cell carcinoma and 21% of adenocarcinomas are located in tissues, whereas 15.7% of squamous cell carcinomas and 25.4% of adenocarcinomas are located in the blood (15). Due to its origin in the bloodstream, adenocarcinoma has multiple primaries, with EGFR mutations associated with HPV (25, 26). The amount of HPV present in the bloodstream that develops into lung cancer carcinoma remains unknown. Squamous cell carcinoma is caused by recurrent respiratory papillomatosis (RRP), which is a direct HPV infection of the respiratory system consisting of type 11 malignant transformations and both types 6 and 11 in other cancer cell types (27, 28). From cohort studies, the incidence of lung involvement in RRP was estimated to be 3.3%. The incidence of cancer involving the lung was 5–16%, mostly type 11. In contrast to most molecular mechanisms in other cancers, HPV types 16 and 18 are unnecessary for oncogenesis. Further research is required to determine the mechanism of oncogenesis other than HPV types 16 and 18.

As the viral load is much lower in lung cancer than in cervical cancer, they must share different molecular mechanisms of oncogenesis and virus subtypes. In cervical cancer, different survival rates were associated with different physical statuses of HPV. For instance, individuals with the episomal form of HPV have a higher survival rate than those with the integrated form (29). This is due to the integration of HPV vectors into the host genome to disrupt gene function, notably the tumor suppressor gene, as well as the expression of E6 and E7. Oncoproteins E6 and E7 are frequently transcribed after the transformation of HPV DNA into the cellular genome in these cancers. The virus-borne E6 and E7 oncoproteins then bind to the tumor suppressor genes p53 and Rb in the host, thus manipulating the cell cycle by disrupting the normal function of the tumor suppressor genes. In our previous studies, we demonstrated that HPV E6 involved in lung tumorigenesis is partially mediated through (1) p53 inactivation (30); (2) upregulation of IL-6, Mcl-1, hTERT, and expression (31, 32); (3) reduced p21 *via* alteration of the p53/DDX3 (dead-box helicase 3) pathway (33); and (4) TIMP-3 inactivation (34). The reduction of p21 facilitates the release of the cyclin A/CDK2 complex (cyclin A/cyclin-dependent kinase 2), which phosphorylates and destroys the active form of pRb (RB transcriptional corepressor 1). On the contrary, the interaction of E7 and pRb leads to the dissociation of the HDAC/pRb/E2F (histone deacetylase/RB transcriptional corepressor 1/E2F transcriptional factor) complex. As a result, HDAC participates in p16INK4 hypermethylation (cyclin-dependent kinase inhibitor 2A), which coordinates with the E2F factor to inactivate tumor suppressor genes. Therefore, cell proliferation genes are activated and tumor progression ensues (35). In addition, HDAC can trigger angiogenesis *via* HIF-1α to induce VEGF and IL-8. The expression of E7 and the inhibition of p53 through the expression of E6 are involved in the regulation of the anti-apoptotic protein Mcl-1, as MCL1 and BCL2 families are apoptosis regulators that increase cell survival by inhibiting apoptosis *via* the PI3K/Akt-(IL-6)-(IL-17) pathway. Further studies suggested that p53 is regulated not only by E6 and E7 but also by E1 and E2 may also be involved (36). E5, a less-discussed oncoprotein, serves as a viroporin at the host cell membrane. E5 serves as an inhibitor of the degradation of epithelial growth factor that facilitates cell growth, prohibiting MHC major histocompatibility class I from floating to the cell membrane, which may elude the immune defense. E5 also prevents hydroxyl peroxidase-induced apoptosis (36).

Even though oropharyngeal and cervical cancers are recognized as the same oncogenic sexual transmission diseases and both are squamous cell epithelium, the pathways of oncogenesis are still different (23). We propose that the oncogenic process of lung adenocarcinoma is also different, and further studies are required to determine the underlying mechanism.

The data in our study were gathered from the National Insurance Database and Cancer Registry Database. However, details of the small incidence subtype, such as small cell and large cell lung cancers, were not provided in the database due to privacy protection policies. One of the main limitations of this study was the unrecognizable subtype and location of HPV. The data on cervical cancer and head and neck cancer were included as the standard of comparison to augment the validity of this study since the study of the HPV association of the two cancers was well established. Similarly, data supporting the association between diabetes mellitus and HPV were also included as the standard of comparison (22). Notably, the HPV incidence rate in our study was 39.44/100,000 person-years for men and 25.54/100,000 person-years for women. This aligned with data from the Cancer Registry Database in 2014, in which the standardized HPV incidence rates were 43.3/100,000 person-years for men and 28.3/100,000 person-years for women.

Although the presence of HPV in lung cancer is a possibility, large-scale full virus subtype tissue screening is required to clarify the role of HPV in various subtypes of lung cancer, such as small cell lung cancer and neuroendocrine cell lung cancers. Furthermore, a study of the virology types of lung cancer to investigate the subtype of HPV in different lung cancers is needed, since most current studies only focus on types 16 and 18. Hence, more pathophysiological studies on other types of HPV are required.

To increase the validity of the study, further population studies for small cell lung cancer are required by collecting a larger database of small and large cell cancers owing to the limited dataset on small cell lung cancer. Another limitation is the distinction between high- and low-risk HPV, as this study included mostly viral warts, unspecific plantar warts, and condyloma accumulates. In addition, the HPV-infected population in this study may be underestimated because individuals with subclinical HPV infection are unlikely to visit clinics, which is a natural limitation of the study population.

Further study designs may include investigation of HPV distribution within the bodies of HPV-infected individuals. The study of the oncogenic mechanisms of adenocarcinoma and squamous cell carcinoma of the lung is also needed because of the different pathways across different HPV and cancer subtypes. For instance, the incidence of HPV-infected lung cancer continues to rise despite the decline of CIS in cervical pre-cancer incidence from 2008 to 2012 for women aged 18–20 years in the United States due to effective vaccination (37). Vaccination effectively decreased HPV types 16 and 18 and genital warts in female individuals aged 13–19 years, while the prevalence trends for anogenital warts varied by age. Nevertheless, overall survival rates of HPV-infected cervical, oral, and lung cancers were better in the past, and more emphasis should be placed on the study of HPV-infected cervical, oral, and lung cancers (29, 38, 39). A high prevalence of EGFR and ALK mutations was found in the lung adenocarcinoma of non-smokers with better survivals, which may benefit from the invention of suitable target therapies, and suggested different oncogenic mechanisms of the cancer itself that call for further investigations (40).

This is the first epidemiological study demonstrating that HPV infection was associated with the occurrence of lung adenocarcinoma, whereas squamous cell carcinoma was mostly associated with COPD. Further validation is warranted to delineate whether there is a causal relationship between HPV infection and lung adenocarcinoma, or simply coexistence.

## Data Availability Statement

The original contributions presented in the study are included in the article/Supplementary Material, further inquiries can be directed to the corresponding author/s.

## Ethics Statement

The studies involving human participants were reviewed and approved by Chung Shan Medical University Hospital IRB. Written informed consent for participation was not required for this study in accordance with the national legislation and the institutional requirements.

## Author Contributions

Conception or design of the work, data analysis, and interpretation: J-YH and FL. Data collection and approval of the manuscript: J-YH, CL, ST, and FL. Drafting the article: CL. Critical revision of the article: ST and FL. All authors contributed to the article and approved the submitted version.

## Funding

This study was funded by the Chung Shan Medical University Hospital CSH-2019-C-005. The English editing and Article Processing Charge are funded by Tungs' Taichung MetroHarbor Hospital TTMHH-C1110072.

## Conflict of Interest

The authors declare that the research was conducted in the absence of any commercial or financial relationships that could be construed as a potential conflict of interest.

## Publisher's Note

All claims expressed in this article are solely those of the authors and do not necessarily represent those of their affiliated organizations, or those of the publisher, the editors and the reviewers. Any product that may be evaluated in this article, or claim that may be made by its manufacturer, is not guaranteed or endorsed by the publisher.
