# Corrigendum: Human papillomavirus is associated with adenocarcinoma of lung: A population-based cohort study

**DOI:** 10.3389/fmed.2022.1045151

**Published:** 2022-09-27

**Authors:** Jing-Yang Huang, Chuck Lin, Stella Chin-Shaw Tsai, Frank Cheau-Feng Lin

**Affiliations:** ^1^Department of Medical Research, Chung Shan Medical University Hospital, Taichung, Taiwan; ^2^Institute of Medicine, Chung Shan Medical University, Taichung, Taiwan; ^3^College of William and Mary, Williamsburg, VA, United States; ^4^Superintendents' Office, Tungs' Taichung Metro Harbor Hospital, Taichung, Taiwan; ^5^National Chung Hsing University, Taichung, Taiwan; ^6^Department of Thoracic Surgery, Chung Shan Medical University Hospital, Taichung, Taiwan; ^7^School of Medicine, Chung Shan Medical University, Taichung, Taiwan

**Keywords:** human papillomavirus, lung cancer, adenocarcinoma, cohort study, big data

In the published article, there was an error in affiliation 1. Instead of “Laboratory of Statistics, Department of Medical Research, Chung Shan Medical University Hospital, Taichung, Taiwan”, it should be “Department of Medical Research, Chung Shan Medical University Hospital, Taichung, Taiwan”.

In the published article, there was an error regarding the affiliation for Jing-Yang Huang. As well as having affiliation 1, he should also have “Institute of Medicine, Chung Shan Medical University, Taichung, Taiwan”.

The authors apologize for these errors and state that this does not change the scientific conclusions of the article in any way. The original article has been updated.

## Publisher's note

All claims expressed in this article are solely those of the authors and do not necessarily represent those of their affiliated organizations, or those of the publisher, the editors and the reviewers. Any product that may be evaluated in this article, or claim that may be made by its manufacturer, is not guaranteed or endorsed by the publisher.

