# Supplementary material for: Human Papillomavirus Is Associated With Adenocarcinoma of Lung: A Population-Based Cohort Study
Source: Front Med (Lausanne). 2022 Jun 30;9:932196. doi: 10.3389/fmed.2022.932196 (PMC9279738; doi:10.3389/fmed.2022.932196)
Supplement: Supplementary file 1 [file Data_Sheet_1.pdf]

10.3389/fmed.2022.932196

Supplementary Table. Adjusted hazard ratio for all, head & neck, and cervical cancers

		Head & Neck Cancer			Cervical Cancer		
		aHR	95% CI	p	aHR	95% CI	p
**HPV**		1.595	1.453-1.749	<.0001*	2.225	2.008-2.464	<.0001*
**Gender**	Male vs Female	5.671	4.999-6.434	<.0001*			
**Age**							
	<20	0.065	0.038-0.109	<.0001*	0.028	0.016-0.048	<.0001*
	20-40	Reference			Reference		
	40-60	4.717	4.128-5.391	<.0001*	1.124	1.001-1.262	0.0481*
	60-80	4.471	3.796-5.266	<.0001*	1.068	0.884-1.291	0.4958
	>=80	3.539	2.687-4.662	<.0001*	0.956	0.633-1.443	0.8292
**Urbanization**							
urban	1	Reference			Reference		
	2	1.145	1.001-1.310	0.0481*	1.004	0.872-1.157	0.9524
	3	1.142	0.975-1.338	0.1000	0.899	0.754-1.072	0.2357
	4	1.251	1.051-1.489	0.0119*	1.141	0.943-1.379	0.1743
	5	1.393	1.010-1.923	0.0435*	0.729	0.450-1.183	0.2007
	6	1.735	1.365-2.206	<.0001*	0.907	0.641-1.283	0.5817
rural	7	1.752	1.352-2.271	<.0001*	0.893	0.613-1.299	0.5532
**Occupation**							
	Officer	0.579	0.460-0.730	<.0001*	0.72	0.565-0.917	0.0077*
	Laborer	Reference			Reference		
	Farmer	1.239	1.066-1.440	0.0053*	1.114	0.920-1.349	0.2685
	Low income	1.238	0.774-1.980	0.3723	1.044	0.590-1.848	0.8825
	Unemployed	1.082	0.954-1.228	0.2214	1.344	1.172-1.542	<.0001*
	Others	0.92	0.608-1.392	0.6927	0.857	0.580-1.266	0.4384
**Co-morbidity**							

10.3389/fmed.2022.932196

Ischemic heart disease	0.849	0.711-1.013	0.0693	0.796	0.591-1.070	0.1310
Hypertension	1.231	1.089-1.392	0.0009*	1.125	0.934-1.355	0.2140
Hyperlipidemia	0.994	0.865-1.142	0.9326	0.808	0.651-1.003	0.0534
Stroke	0.87	0.695-1.090	0.2258	0.922	0.636-1.336	0.6668
Diabetes mellitus	1.452	1.260-1.674	<.0001*	1.109	0.873-1.410	0.3965
Abnormal liver function	1.72	1.504-1.967	<.0001*	1.334	1.067-1.669	0.0116*
Peptic ulcer	1.153	1.005-1.323	0.0421*	1.19	0.994-1.425	0.0581
GI bleeding	1.379	0.948-2.008	0.0932	1.422	0.755-2.677	0.2754
Renal failure	1.163	0.704-1.922	0.5559	2.725	1.235-6.009	0.013*
Chronic kidney diseases	0.994	0.661-1.495	0.9758	1.001	0.497-2.016	0.9984
Gout	0.932	0.779-1.115	0.4436	1.109	0.717-1.716	0.6411
COPD	1.059	0.847-1.324	0.6155	1.109	0.738-1.665	0.6193

COX Regression. *p<0.05.

10.3389/fmed.2022.932196

S1. Cervical can cancer, P<0.001

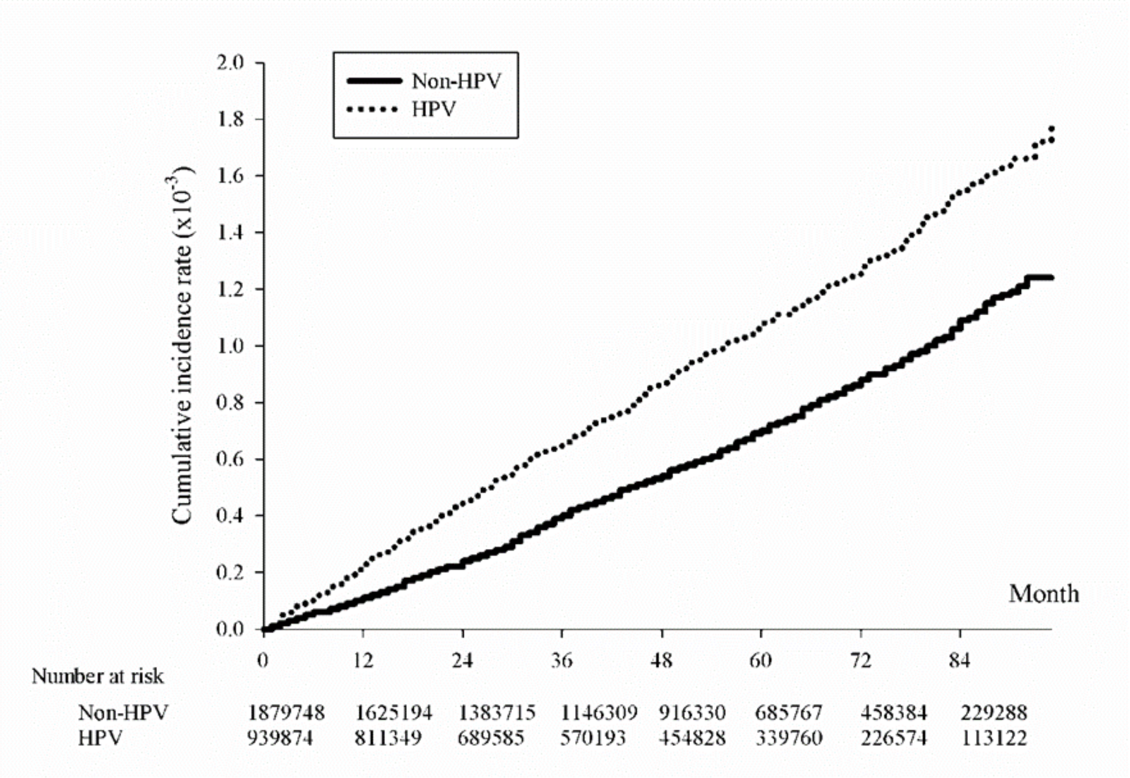

Supplementary Figure 1. Kaplan-Meier curve of cervical cancer cumulative incidence rates with and without HPV infection

10.3389/fmed.2022.932196

S2. Head and neck cancer. P<0.001

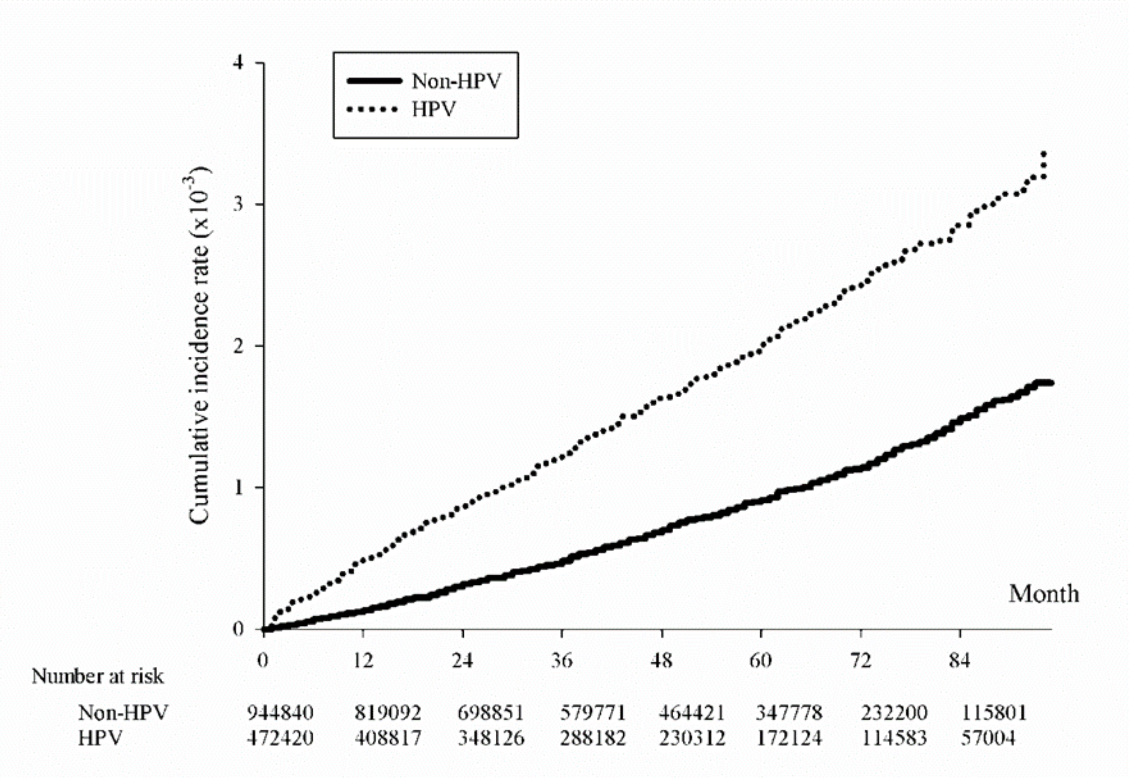

Supplementary Figure 2. Kaplan-Meier curve of head and neck cancer cumulative incidence rates with and without HPV infection
